# Does dietary fat intake influence oocyte competence and embryo quality by inducing oxidative stress in follicular fluid? 

**Published:** 2013-12

**Authors:** Ashraf Kazemi, Fatemeh Ramezanzadeh, Mohammad Hosein Nasr-Esfahani, Ali Akbar Saboor Yaraghi, Mehdi Ahmadi

**Affiliations:** 1 *Nursing and Midwifery Care Research Center, Faculty of Nursing and Midwifery, Isfahan University of Medical Sciences, Isfahan, Iran.*; 2 *Vali-e-Asr Reproductive Health Research Center, Imam Khomeini Hospital Complex, Tehran University of Medical Sciences, Tehran, Iran.*; 3 *Department of Reproduction and Development, Reproductive Biomedicine Center, Royan Institute for Animal Biotechnology, ACECR, Isfahan, Iran. *; 4 *Department of Nutrition and Biochemistry, School of Public Health, Tehran University of Medical Sciences, Tehran, Iran.*; 5 *Isfahan Fertility and Infertility Center, Isfahan, Iran.*

**Keywords:** *Dietary Fat*, *Oxidative stress*, *Follicular fluid*, *Oocyte competence*, *Embryo Quality*, *Malon-de-aldeid*, *Total antioxidant capacity*

## Abstract

**Background:** Fat-rich diet may alter oocyte development and maturation and embryonic development by inducing oxidative stress (OS) in follicular environment.

**Objective: **To investigate the relationship between fat intake and oxidative stress with oocyte competence and embryo quality.

**Materials and Methods:** In observational study follicular fluid was collected from 236 women undergoing assisted reproduction program. Malon-di-aldehyde (MDA) levels and total antioxidant capacity (TAC) levels of follicular fluid were assessed as oxidative stress biomarkers. In assisted reproduction treatment cycle fat consumption and its component were assessed. A percentage of metaphase ΙΙ stage oocytes, fertilization rate were considered as markers of oocyte competence and non-fragmented embryo rate, mean of blastomer and good cleavage (embryos with more than 5 cells on 3 days post insemination) rate were considered as markers of embryo quality.

**Results:** The MDA level in follicular fluid was positively related to polyunsaturated fatty acids intake level (p=0.02) and negatively associated with good cleavage rate (p=0.045). Also good cleavage rate (p=0.005) and mean of blastomer (p=0.006) was negatively associated with polyunsaturated fatty acids intake levels. The percentage of metaphase ΙΙ stage oocyte was positively related to the TAC levels in follicular fluid (p=0.046). The relationship between the OS biomarkers in FF and the fertilization rate was not significant.

**Conclusion:** These findings revealed that fat rich diet may induce the OS in oocyte environment and negatively influence embryonic development. This effect can partially be accounted by polyunsaturated fatty acids uptake while oocyte maturation is related to TAC and oocytes with low total antioxidant capacity have lower chance for fertilization and further development.

## Introduction

The follicular fluid (FF) surrounding the oocytes, plays a critical role in constituting a complex ovarian microenvironment that influences oocyte quality and thereby affects assisted reproduction outcomes. Oxidative stress (OS) refers to overproduction of free radicals and reactive oxygen species which are generated as part of a normal cellular metabolism and/or a deficit in the capacity of antioxidant defense systems, leading to oxidation of biologic macromolecules (1). Several studies have reported that OS in antral follicle has a deleterious effect on the developmental competence of human oocytes, but many studies have provided evidence that OS in FF does not affect the assisted reproduction outcomes (2-5). Although these studies were well designed, they did not consider the environmental factors that can influence OS in FF which in turn affect the assisted reproduction outcomes.

Some external factors, related to life-style, may damage the oocyte competence through an overload of reactive oxygen species. Among these factors, it is known that fat-rich diet is associated with increased serum cholesterol and triglyceride levels and is linked to induction of OS. It has been shown that increased circulating level of low-density lipoproteins induces OS in endothelial cells and such phenomenon lead to systemic pathological process, but limited data are available on the effect of dietary fat intake on the OS in follicular environment and assisted reproduction outcomes (6-9).

Therefore, a change in follicular lipids due to high dietary fat intake may affect the peroxidative process in antral follicle. However, it must be noted that due to the unique characteristics of FF, its composition is different from blood and for this reason the effect of dietary fat on OS in FF needs to be further explored (10, 11). Considering that non-enzymatic antioxidants in the FF mainly originated from serum, therefore, decreased antioxidants in plasma, due to high fat dietary intake may prone the environment of FF to peroxidative stress. 

Therefore, to demonstrate the impact of fat intake on the oocyte competence due to induced-OS in FF, the relationship between the dietary fat intake level and lipid peroxidation product in FF, in addition to the relationship between dietary fat intake and oocyte competence, were evaluated. Since, body mass index (BMI), physical activity and smoking status may influence both the OS in FF and oocyte competence, they were referred to as influential parameters.

## Materials and methods

An observational study included 240 consecutive series of in vitro fertilization/intra-cytoplasmic sperm injection (IVF/ICSI) candidates from July 2010 to April 2011 was conducted at Isfahan Fertility and Infertility Center. Institutional Review Board and the Ethics Committee of Tehran University of Medical Sciences approved the study. Informed consents were obtained from all subjects. The inclusion criterion was being aged from 18-40 years old and the exclusion criteria were male factor infertility according to World Health Organization criteria, systemic disease and considerable change in dietary regimen over the previous three months and during assisted reproduction cycle (12).

**Questionnaires**

At the start of the medication cycle, characteristics of patients and smoking status were recorded. A validated semi-quantitative food frequency questionnaire (FFQ) including 168 food items was used to measure the calorie intake, total dietary fat, and its components including saturated FAs (SFAs), mono-unsaturated FAs (MUFAs), and poly-unsaturated FAs (PUFAs) over the previous three months (13). Depending on the portion size, the frequency of food intake was recorded in times per day, week and month or never. For all the main food items in the FFQ, the frequency per day was multiplied by the amount consumed, depending on the portion size, to compute the total amount consumed per day. 

Nutritionist software version IV (Nutritionist IV, Version 3.5.2) was used to calculate the daily energy and fat component. The USDA food composition table was used for most items (USDA, Release 11, 1994). Physical activity was measured using the original International Physical Activity Questionnaires. This questionnaire assessed the energy expenditure in total, by moderate and vigorous intensity and by using metabolic equivalent (MET) values and formula for computation of MET-minutes (14, 15). BMI was calculated by dividing weight in kilograms by height in meters squared.

**Ovulation Induction**

The long protocol, involving GnRH agonist and hMG/Gonal F administration, was consistent and follicular maturation was monitored by ultrasound examination. In brief, daily subcutaneous GnRH agonist (Superfact, Hoechst, Germany) was started in the mid-luteal phase of the previous cycle and gonadotropin stimulation of the ovaries commenced on day 2 of last menstrual period, for 12-14 days when transvaginal sonography showed absence of follicles/cysts. For all patients, gonadotropin therapy was initiated with a daily dose of 100-300 IU of either with rFSH (Gonal F, Serono, Rockland, MA, USA, Follistim, Organon, and Roseland, NJ) and/or hMG (Menogon, Ferring, Suffern, NY) which was adjusted according to follicular response. Ovulation was triggered with 10,000 IU hCG (Pregnyl, Organon), when dominant follicles reach a follicular size of around 18 mm. 

36 hours later, the oocytes were collected transvaginally and used for IVF or ICSI procedure. Despite exclusion criteria of male factors infertility, as routine protocol of Isfahan Fertility and Infertility Center, portion of oocyte were inseminated through ICSI procedure. At oocyte retrieval, fluid from an average of one to 5 follicles of 16 mm diameter or larger was pooled and centrifuged at 300 rpm for 17 minutes, and 2 ml of the supernatant was stored at -70^o^C, for maximum 2 weeks, until analyzed for malon-di-aldehyde (MDA) and total antioxidant capacity (TAC). The samples without oocyte or contaminated with RBC were discarded. The oocytes were scored for presence or absence of germinal viscule and first polar body. Oocytes were considered fertilized when two pronuclei was observed 17-19 hours post insemination. Numbers of blastomer were defined and percentage of embryos without fragmentation was calculated. 

Embryos with more than 5 cells on 3 days post insemination were considered as good cleaved embryos. In ICSI cycles, the percentages of MII stage were calculated by dividing the number of oocytes with first polar body by number of oocytes that were assessed. In IVF/ICSI cycles the percentages of MII stage were calculated by dividing the number of oocytes presenting at least one polar body by the number of oocytes that were assessed. Embryos with less than 5% fragmentation were considered as non-fragmented and percentage of these embryos was calculated.

**Laboratory analysis**

Aliquots of the FF were thawed at room temperature and follicular fluid lipid peroxidation was assessed by MDA assay according to Oral *et al* and TAC was assessed by enhanced chemiluminescence assay as described previously (16, 17). All the samples were protected from direct sunlight.

**Statistical analysis**

Statistical analysis was performed using SPSS 13.00 (Chicago, IL, USA). The normality of the distributions of fat and its components intake levels were assessed by Q-Q plots and found to be skewed. Log transformation improved normality and the values were used throughout the analysis. Descriptive analyses were performed using mean and standard deviation. The data were analyzed using linear regression (adjusted for age, BMI, physical activity, smoking status, calorie consumption and etiology of infertility) and t-test. The p-value of less than 0.05 was considered to be significant. 

## Results

In total, 240 couples participated in the study. Due to ovarian hyper stimulation syndrome, four subjects were excluded from the study. Profiles of subjects are presented in table I. Associations between the OS biomarkers in FF and fat components intake adjusted for age, physical activity, BMI, passive smoking status, etiology of infertility and calorie intake are presented in table II. Among fat components PUFA level intake significantly was associated with the MDA levels in FF. The association between the total fat intake and the MDA levels in FF was not significant. But, the mean value of MDA levels in FF in women with the total fat intake of greater than and lower/equal than/to 40 gr/d was significantly different (1.0±0.29 vs. 0.89±0.22; p=0.02). 

Relationships between the fat components and TAC level in FF were not significant. Association between the oocyte competences and embryo quality markers with biomarkers of stress oxidative in FF, adjusted for age, BMI, physical activity, smoking status and etiology of infertility are presented in table III. Patients’ age was significantly and negatively associated with oocyte maturation rate (% MII oocyte). After adjustment for influential variables, the MII stage oocyte rate was associated with an increase in the TAC levels in FF. The relationships between the OS biomarkers in FF and the fertilization rate, non-fragmented embryo rate and mean of blastomer were not significant. 

The good cleavage rate was negatively related to the MDA levels in FF. Association of the oocyte competence parameters and the fat components intake adjusted for influential factors revealed that the total fat intake and calorie intake from fat were negatively associated with percentage of non-fragmented embryo. Also, the mean of blastomer and the good cleavage rate were significantly and negatively related to the PUFA intake (Table III). 

Although, table III shows significant negative relations between the level of PUFA intake and MDA level in FF with good cleavage rate, when using linear regression analysis, the result revealed that the effect of PUFA intake level on good cleavage rate was dependent on MDA levels in FF (B:-0.28, CI:-25.55-25.98).

**Table I:** Characteristics of subjects

**Variables **				**Mean (SD) or %**
Age (year)				31.54 (6.20)
PA (METs-minutes-day)				31.82 (5.51)
BMI (kg/m^2^)				26.6 (4.3)
Active smoker (%)				0.00
Passive smoker (%)				30.1
Etiology of infertility (%)				
	PCOS			29.7
	Endometriosis			18.2
	Anovulation			15.3
	Other etiologies			36.8
Total calorie intake (kcal)				2167.8 (717.2)
Calorie as fat (%)				30.67 (8.63)
Fat components				
		Total fat (gr/day)		72.6 (31.93)
		Sat (gr/day)		20.2 (6.9)
		MUFA (gr/day)		27.1 (10.5)
		PUFA (gr/day)		23 (16.8)
MDA (µ mol/lit)				0.98 (.28)
TAC (molar Trolox equivalents)				1987.73 (354.08)
Oocyte competence parameters				
			MII stage oocyte rate	78.7 (27.5)
			Fertilization rate	62.6 (30.1)
			Non-fragmented embryo rate	44.81 (36.4)
			Mean of blastomer	6.13 (2.65)
			Good cleavage rate	71.5 (36.6)

PA: physical activity.

BMI: body mass index.

PCOS: Poly cystic ovaries syndrome.

Sat: Saturated fatty acids.

MUFA: Mono-unsaturated fatty acids.

PUFA: Poly-unsaturated fatty acids.

MDA: Malon-di-aldehyde.

TAC: Total antioxidant capacity

**Table II:** Relation between fat consumption and its components with oxidative stress in FF adjusted for age, physical activity, BMI and etiology of infertility

		**MDA**				**TAC**			
		**B**	**CI**		**Sig**	**B**	**CI**		**Sig**
Calorie as fat (%)		0.01	-0.02	0.01	0.08	1.83	-3.5	7.16	0.34
Fat component:									
	Total fat (g/d)	0.30	-0.17	1.26	0.12	-21.72	-148.19	816.67	0.40
	Sat. FA (g/d)	-0.38	-0.92	0.15	0.23	-292.56	-1394.14	809.02	0.55
	MUFA (g/d)	-0.22	-0.64	0.21	0.15	842.27	-99.27	1783.81	0.46
	PUFA (g/d)	0.48	0.08	0.89	0.02	-501.64	-2087.10	1083.83	0.35

BMI: body mass index.

Sat: Saturated fatty acids.

MUFA: Mono-unsaturated fatty acids.

PUFA: Poly-unsaturated fatty acids.

MDA: Malon-di-aldehyde.

TAC: Total antioxidant capacity.

CI: confidents interval.

Regression test were used.

**Table III:** Relation between fat consumption and its component with oocyte competence and embryo quality adjusted for age, physical activity, BMI and etiology of infertility

	**MII stage oocyte R.**			**Fertilization R.**			**None fragmented embryo R.**			**Mean of blastomer**			**Good cleavage R.**		
	**B**	**CI**		**B**	**CI**		**B**	**CI**		**B**	**CI**		**B**	**CI**	
MDA	0.26	-10.1	10.6	-0.11	-0.64	0.42	-7.7	-26.2	10.8	-0.81	-2.02	0.42	-16.6^†^	-33.2	0-.02
TAC	0.03^†^	0.01	0.04	-0.01	-0.01	0.01	0.01	-0.01	0.02	0.01	-0.01	0.01	-0.01	-0.02	0.01
Calorie as Fat (%)	-15.4	-84.1	53.4	-3.6	-80.1	72.8	-.84†	-1.5	-.2	-.01	-.18	.15	29.8	-61.2	121
Fat component															
Total fat (g/d)	37.6	-2.4	77.6	17.4	-27	61.9	-48.7†	-89.2	-8.1	-0.89	-13.4	11.6	33.2	-19.8	86.1
Sat. FA (g/d)	31.7	-3.5	66.9	31.1	-8	70.2	-10.8	-64.2	42.3	2.2	-1.5	5.9	21.2	-25.4	67.7
MUFA (g/d)	-26.1	-66.4	14.1	-8.4	-53.1	36.4	25.1	-33	83.5	2.2	-1.3	5.6	14.9	-38.4	68.2
PUFA (g/d)	-34.2	-70.8	2.5	-26.9	-67.6	13.8	3.9	-35.8	42.9	-5.9††	-9.6	-2.2	-65††	-115	-18

^†^p<0.05, ^††^p<0.01;

BMI: body mass index.

MDA: Malon-di-aldehyde.

TAC: Total antioxidant capacity.

Sat: Saturated fatty acids.

MUFA: Mono-unsaturated fatty acids.

PUFA: Poly-unsaturated fatty acids.

R.: rate

Regression test were used.

## Discussion

To answer the question “whether dietary fat intake influences oocyte competence by inducing OS in FF”, three objectives were followed in this study including the relations between the dietary fat intake and OS in FF, the OS in FF and oocyte competence, and the fat intake and oocyte competence. Initially, the result of this study revealed that the relation between the OS in FF and total fat intake was not linear. However, in the women with fat intake of over 40 gr per day, the MDA level was significantly higher than the women with lower fat intake. Therefore, it could be concluded that the oocytes of individuals with dietary fat intake of over 40 grams per day are prone to OS and this might be due to high PUFA intake. 

Despite the positive role of PUFAs intake on health status at systemic level, which is orchestrated through the balance of LDL/DHL profile, PUFAs are considered as suitable substrates for production of ROS, mainly lipid peroxidation; the byproduct of which is MDA (6, 18-20). Therefore a positive relation between the MDA levels in FF and PUFAs intake level and the inverse relation between the MDA levels in FF and ICSI/IVF outcomes, such as mean of blastomer and good cleavage rate, suggest that PUFA may play its role by increasing lipid peroxidation byproduct which in turn may negatively affect ICSI/IVF outcome. 

These observations are consistent with previous reports which showed that addition of linoleic acid, to bovine and mice cumulus-oocyte complexes, significantly inhibited cumulus cell expansion and retarded development of the oocytes to the metaphase II (MII) stage in a dose-dependent manner and adversely affected its subsequent development; however this effect is reversible (21). In contrary to our results and these reports, Bilby *et al* showed that there was feeding dairy cow with polyunsaturated FAs, as compared to mono-unsaturated FAs had no positive effect on oocyte quality, and its subsequent embryo development (22). 

These inhibitory effects may be due to oxidation of PUFA in the cellular membrane which would impair membrane function and structural integrity, and would decrease membrane fluidity which can inactivate membrane bound enzymes or hormonal interaction at membrane level which is important in follicular endocrine function (23). Indeed, Wakefield *et al* revealed that exposure of the follicle to n-3 rich PUFA due to maternal dietary of high n-3 PUFA, alters mitochondrial distribution and calcium levels and increases the production of reactive oxygen species (24). 

Also, our data showed the negative association between the total fat intake and non-fragmented embryo rate. This observation is consistent with a previous report by Jungheim *et al* which showed an association between poor oocyte morphology and increased total fat concentration in FF in individuals undergoing assisted reproduction (25). They further showed that increased serum FFA is associated with decreased chance of pregnancy, despite low association between FF and serum FFA. 

The second objective of this study was to evaluate the relation between OS in FF and oocyte competency. The MDA levels showed a negative correlation with good cleavage rate, suggesting that oocytes developed in OS have lower chance to cleave. Consistent with our findings, previous studies reported that elevated reactive oxygen species levels were seen in FF containing embryos of poorer morphology or negative association observed between cleavage rate with protein oxidation levels (2, 3).

Despite our results and others, not all studies in this field have demonstrated a relation between antioxidant defense capacity and oocyte maturation and in regard to this, some researchers have suggested that antioxidants including vitamins A, C and E, despite their antioxidant capacity, may exert their effect on oocyte maturity through other routes (5, 26-28). This possibility may account for lack of relation between oocyte maturity and MDA level, suggesting not all the antioxidants are required as defense mechanism to lipid peroxidation, but the antioxidants are required in the innate process of oocyte maturation, such as glutathione required for sperm decondensation (29). 

This could be a reason that the MDA, rather than TAC depletion, is considered as a better marker of oxidative stress (30). However, despite these reports, it was interesting to note that TAC only showed a positive relation with oocyte maturation rate. This would suggest that oocytes that do not receive adequate antioxidant have lower chance to complete meiosis, and therefore, participate in the process of fertilization and development. Indeed, dominate follicles have higher degree of angiogenesis and therefore, are more likely to receive higher amount of antioxidant from systemic circulation. This observation is consistent with previous report, suggesting that TAC level in FF of immature oocytes would be lower compared to MII oocyte in PCOS patients (31). 

Glutathione, as source of antioxidant is essential to oocyte health and play an important role in sperm decondensation post sperm penetration into oocyte (27, 32, 33). The negative effect of PUFA on reproductive outcomes may be accounted at two levels. On one side the individuals may have excess amount of PUFA in their membrane which prone their cells to OS, or on the other side, they have low level of antioxidant intake, like vitamin E, which are essential for inhibition of propagation lipid peroxidation at membrane level. Indeed, Roberts et al showed that systemic OS was accompanied by up regulation of NAD(P)H oxidase, pointing to increased reactive oxygen species production capacity, and down regulation of the key enzymes in the antioxidant defense system (34). 

Furthermore, metabolic and hormonal pathways, which could influence oocyte quality can be affected by fat rich diet and it has been shown that foods high in fat are rich in advanced glycation end-products and there accumulation in plasma may increase OS in plasma and FF which is derived from the plasma (8, 35-44). Indeed, major cause of macromolecular damage in follicles is suggested to be advanced glycation end-products (45). Another mechanism which may explain the reduction of oocyte competence in these individuals, is that, the high fat intake is accompanied by insulin resistance, which may induce OS in follicular microenvironment and this effect might also be mediated via advanced glycation end-products, the production of which might also be accounted by thermoxidised vegetable oil as a major source of PUFA widely used in developing countries (41, 46, 47). 

Despite the aforementioned possibilities, excessive intracellular lipids storage, in term of high lipid droplets within oocyte is considered as a negative factor and has been associated with suboptimal mitochondrial function, hampering the quality and viability of the embryo, especially during cryopreservation (48, 49). Another alternative route through which fat may negatively affect embryo development is via alteration of the oviductal luminal fluid lipids, which was not possible to assess in this study (50). Another observation was the negative association between the percentages of non-fragmentation embryo and fat as source of calorie. This could be explained by the aforementioned deleterious effects of PUFA, but this effect might not be a direct effect, since PUFA showed no significant elation with this parameters. 

## Conclusion

In conclusion the results of this study suggest that, fat intake over 40 gr per day increases the level of lipid peroxidation as a result of oxidative stress, assessed by MDA. However, among fat component only PUFA showed a significant relation with MDA, suggesting that increased uptake of PUFA prone cumulus oocyte complex to oxidative stress via PUFA which in turn affects the mean of blastomer and quality of embryo. Unlike PUFA, calorie intake improved these parameters and also reduced the embryo fragmentation rate. The results also revealed that lack of antioxidant capacity within follicle would reduce the maturation ability of the oocyte to reach MII, and thereby it has no influence on latter stage since these oocytes are not used for insemination. Furthermore, since embryo quality is affected by both male and female components, lack of assessment of this factor is considered as a limitation in this study.
